# Rotavirus Vaccine Administration Errors — United States, 2006–2013

**Published:** 2014-01-31

**Authors:** Beth F. Hibbs, Elaine R. Miller, Tom Shimabukuro

**Affiliations:** 1 Immunization Safety Office, Division of Healthcare Quality and Promotion, National Center for Emerging and Zoonotic Infectious Diseases, CDC

Two live rotavirus oral vaccines, RotaTeq (RV5) (Merck & Co., Inc.) and Rotarix (RV1) (GlaxoSmithKline Biologicals) (Figure), are approved for prevention of rotavirus gastroenteritis (1) and recommended at ages 2, 4 (RV5/RV1), and 6 (RV5) months by the Advisory Committee on Immunization Practices. Because most childhood vaccines are injectable, vaccination providers might have less experience administering oral vaccines. To assess that hypothesis, CDC searched for reports to the Vaccine Adverse Event Reporting System (VAERS) (2) of rotavirus vaccine administration errors involving injection and eye splashes in the United States during the period January 1, 2006–August 1, 2013. A total of 66 reports were found.

There were 39 reports of administration by injection (33 for RV1 and six for RV5). This included a cluster of six reports involving RV1 by a nurse who did not receive proper training or read the package insert. Nineteen of the 39 reports (49%) documented an adverse event; irritability (seven cases) and injection site redness (five) were the most commonly reported adverse events. Thirty of 39 reports (77%) did not have an explanation for the error; for those that did, reasons included misinterpreting package insert instructions, confusing the RV1 oral applicator syringe with a syringe for injection, confusing the RV1 vial with a vial used for injectable vaccine, inadequate training, and not reading the package insert.

There were 27 reports of eye splashes. In 21 cases, infants coughed, sneezed, or spit vaccine into the eyes of vaccination providers (17), parents (one) or themselves (three). Nonserious adverse events consistent with minor eye irritation were described in 21 of the 27 reports.

As a passive surveillance system, VAERS might capture only a small fraction of vaccine administration errors. However, with approximately 55 million doses (3) distributed, these incidents appear to be rare. Vaccination providers should follow instructions in package inserts regarding proper administration. An injected dose of RV1 or RV5 is not considered a valid dose, and a properly administered oral replacement dose should be given within the appropriate age and dosing schedule. Vaccination providers should be aware of the potential for eye splashes. Vaccine should be administered gently inside the cheek to minimize coughing, sneezing, and spitting. If a child does regurgitate, spit out, or vomit during or after administration, administration of a replacement dose is not indicated (1). Administration errors are largely preventable with proper education and training.

## Figures and Tables

**FIGURE:**
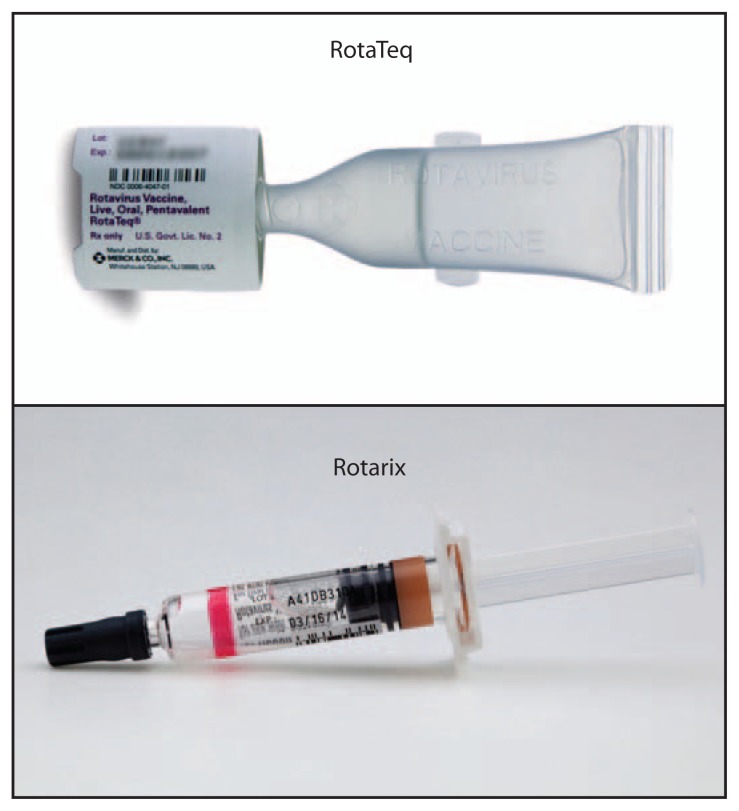
Two live rotavirus oral vaccines (RotaTeq and Rotarix)^*^ Photos/Merck & Co., Inc. (RotaTeq) and GlaxoSmithKline Biologicals (Rotarix) ^*^ During the period January 1, 2006–August 1, 2013, a total of 66 reports of rotavirus vaccine administration errors were submitted to the Vaccine Adverse Event Reporting System, including 39 reports of administration by injection (six for RotaTeq and 33 for Rotarix), of which nine reports included an explanation for the error, which included the following: misinterpreting package insert instructions, confusing the Rotarix oral applicator syringe with a syringe for injection, confusing the Rotarix vial (not pictured) with a vial used for injectable vaccine, inadequate training, and not reading the package insert.
